# Supplementary material for: Active learning for nonparametric multiscale modeling of boundary lubrication
Source: Sci Adv. 2025 Sep 12;11(37):eadx4546. doi: 10.1126/sciadv.adx4546 (PMC13155564; doi:10.1126/sciadv.adx4546)
Supplement: Supplementary file 1 — Figs. S1 to S6 [file sciadv.adx4546_sm.pdf]

Science Advances
AAAS

Supplementary Materials for

Active learning for nonparametric multiscale modeling of boundary lubrication

Hannes Holey *et al.*

Corresponding author: Hannes Holey, hannes.holey@unimi.it; Lars Pastewka, lars.pastewka@imtek.uni-freiburg.de

Sci. Adv. **11**, eadx4546 (2025)
DOI: 10.1126/sciadv.adx4546

This PDF file includes:

Figs. S1 to S6

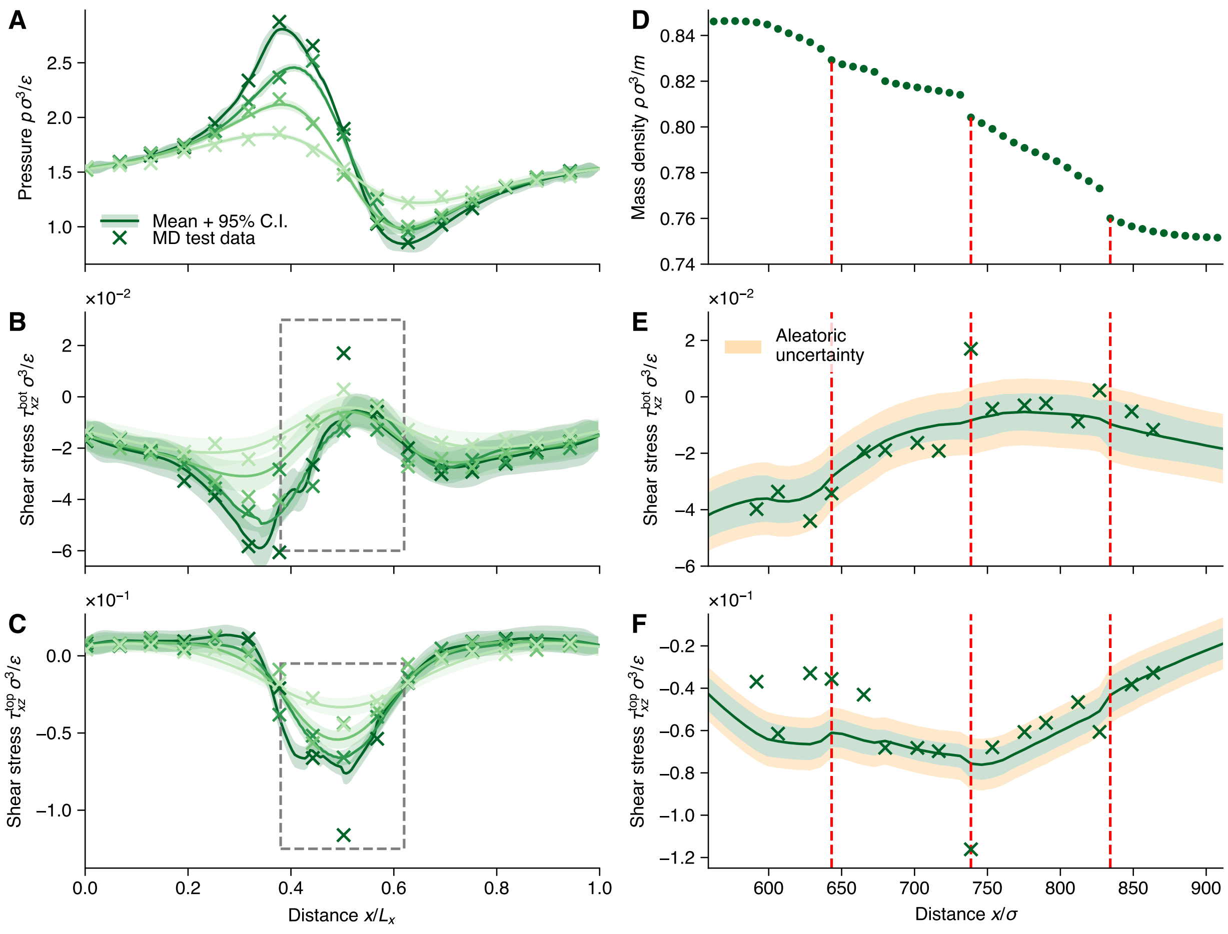

Figure S1: **Comparison of stress profiles from Fig. 2 with additional molecular dynamics (MD) test data that was not used for fitting the Gaussian process (GP). A** Pressure predictions (same as Fig. 2D of the main text), **B** shear stress predictions at the bottom wall (same as Fig. 2E of the main text), and **C** shear stress predictions at the top wall (same as Fig. 2F of the main text) but with additional MD test data. The dashed gray box in **B** and **C** highlight the zoomed in regions shown in **E** and **F**, respectively. **D** Density profile for the narrowest gap ($h_0 = 5.88\sigma$) in the center of the domain. Individual points correspond to the density within a finite volume element. The red dashed lines indicate jumps or kinks in the density profile. **E** Zoom into the shear stress prediction at the bottom wall in the center of the domain and additional MD test data. **F** Same as **E** but for the top wall. Outliers coincide with locations of density kinks and jumps, indicating their relation to layering transitions.

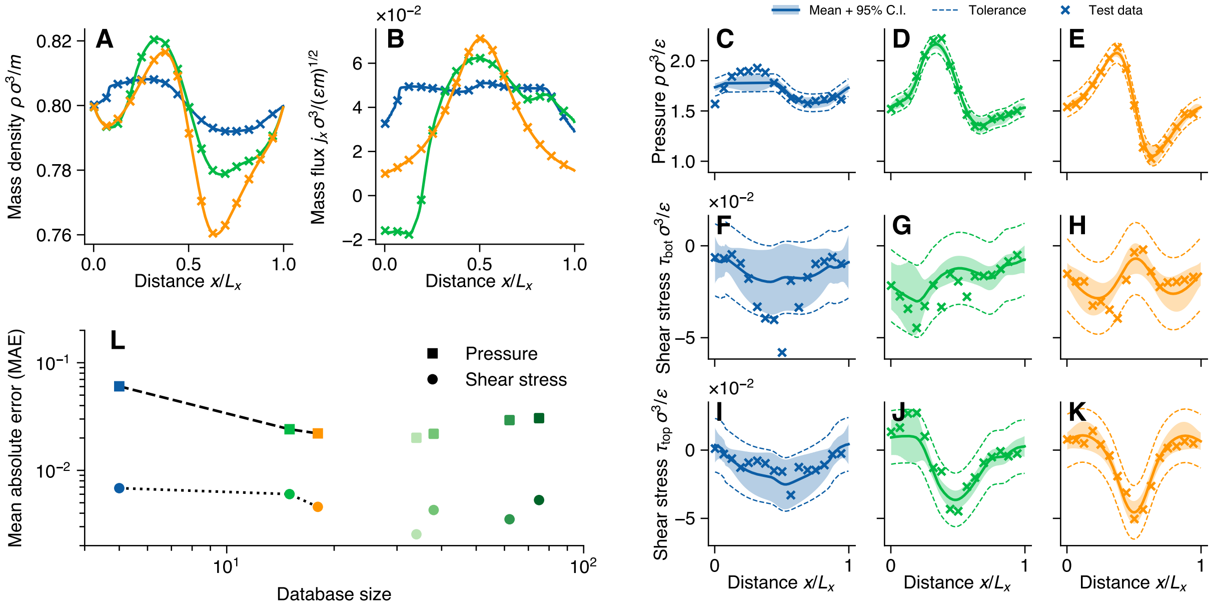

Figure S2: **Error estimates at different time steps based on molecular dynamics (MD) test data. A** Density profiles at three time steps: blue (step 79), green (step 240), and orange (step 876). Crosses mark equidistant points in the x domain which determine the inputs for additional MD simulations as test locations. **B** Mass flux profiles at three time steps. **C–E** Pressure predictions including the Gaussian process (GP) posterior variance and the uncertainty tolerance. Crosses indicate the output of the MD test data. **F–H** Shear stress prediction and MD measurement at the bottom wall. **I–K** Shear stress prediction and MD measurement at the top wall. **L** Mean absolute error (MAE) for pressure and shear stress prediction based on the MD test data. Test data computed for three different time steps within a single run are connected with dashed or dotted lines. MAE values for the steady state profiles shown in Fig. 2 of the main text are shown as green symbols.

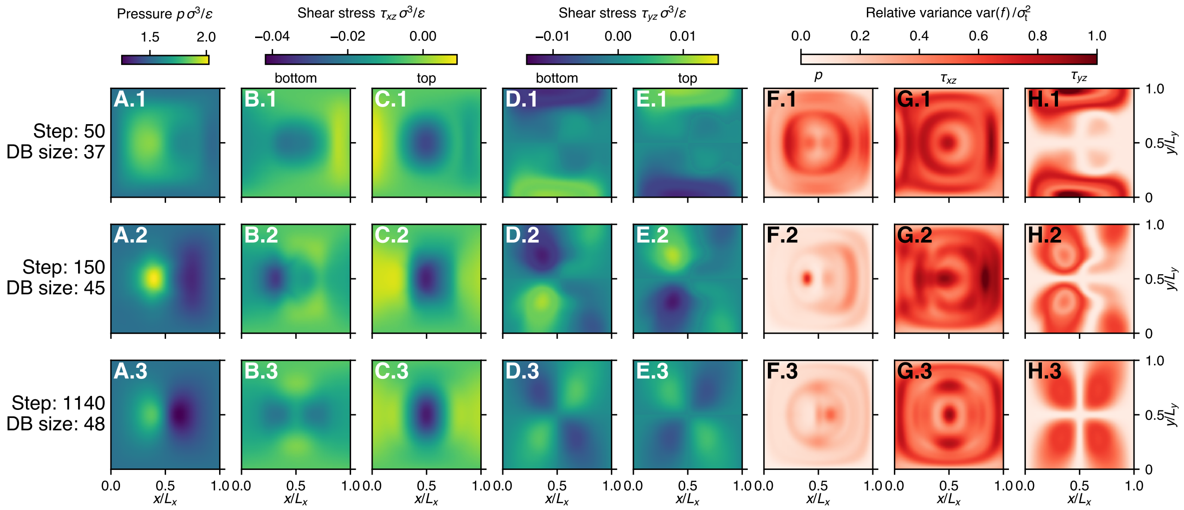

Figure S3: **Evolution of pressure and shear stress predictions during a training run on a two-dimensional example problem mimicking the flow around a single asperity.** Column **A** illustrates the Gaussian process (GP) posterior mean pressure predictions for three different simulation time steps and database (DB) sizes. The minimum gap height in the center of the domain is 8.82σ. The first row corresponds to an initial stage of the simulation, with a prediction based on data from 37 molecular dynamics (MD) runs. The second row corresponds to a time frame one hundred steps later with 45 MD runs, and the third and last row shows the converged solution after 1140 steps and 48 MD runs. Columns **B** and **C** show the shear stress predictions of the GP in the shearing direction τ_{xz} at the bottom and top wall, respectively, for the three time frames. Accordingly, the shear stress perpendicular to the shearing direction τ_{xz} is shown in columns **D** and **E**. The last three columns **F**, **G**, and **H**, show the posterior variance of the GP regression models for pressure and the two shear stress components, respectively. The variance is normalized by the corresponding uncertainty tolerance which is $(\sigma^0_{\mathrm{t},p})^2 = 2.5 \times 10^{-4}\epsilon^2\sigma^{-6}$ for the pressure and $(\sigma^0_{\mathrm{t},\tau})^2 = 1.25 \times 10^{-5}\epsilon^2\sigma^{-6}$ for the shear stress, i.e., the largest refinement level used for the single asperity training calculations. Hence, dark red regions indicate low predictive confidence and brighter regions correspond to regions with relatively high confidence.

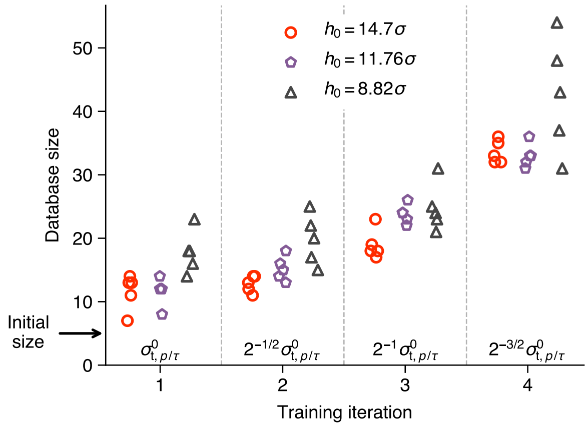

Figure S4: **Size of the training database for the single asperity geometry.** Same as Fig. 4A of the main text but for two-dimensional simulations.

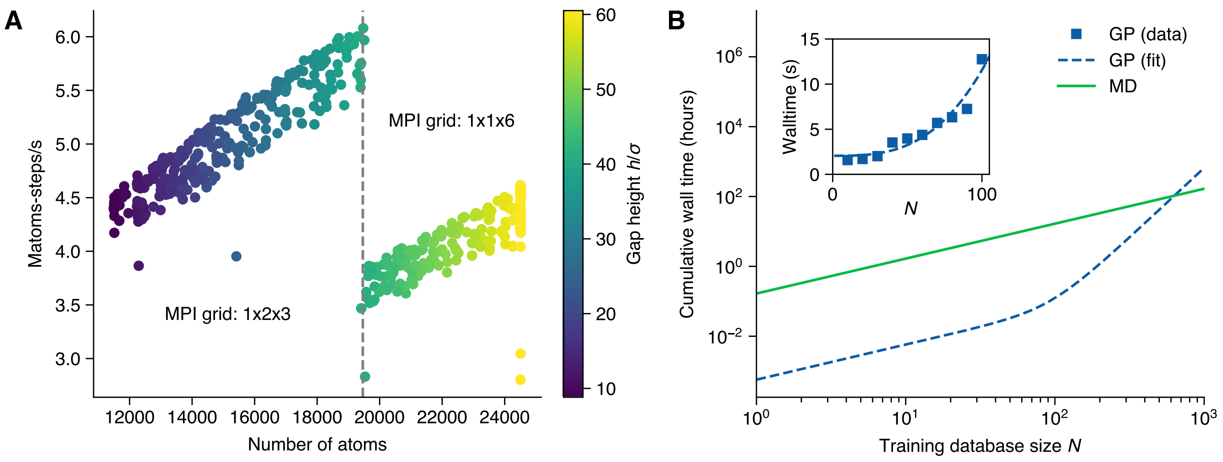

Figure S5: **Performance evaluation for molecular dynamics (MD) runs and Gaussian process (GP) training. A** Million atom time steps per second vs. number of atoms, which increases with the gap height highlighted in color. Due to unfavorable domain partitioning (LAMMPS default) for systems with gap heights larger than 40σ or more than 19 500 atoms, the performance drops notably for larger systems. This issue is fixed in forthcoming versions of our code. Outliers are likely due to accidental oversubscription of the available CPUs. **B** Approximate scaling of the cumulative wall time with the training database size for MD runs and GP training. A single GP training scales with the cube of the training database size. We measured wall times for individual training steps including hyperparameter optimization for up to 100 training points (inset). From a fit to the data (with fit function $f(N) = A + BN^3$) shown in the inset, we compute the cumulative sum of the GP training wall time assuming that the database is built from scratch within a single run (dashed line). Although the MD cumulative wall time scales only linearly (solid line), MD will stay the computational bottleneck for databases with less than 600 points. More sophisticated MD models (e.g. interatomic potentials) will enhance this trend, i.e. shift the MD curve upwards.

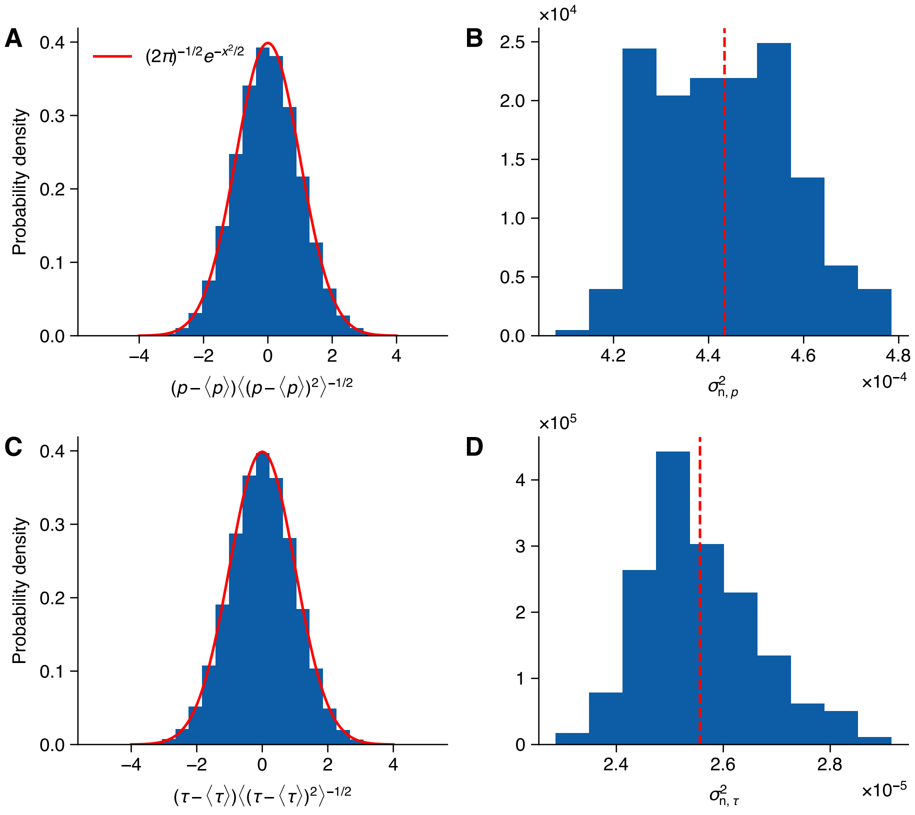

Figure S6: **Noise analysis for the stress components sampled from molecular dynamics (MD) simulations. A** Probability distribution of the standardized pressure from 248 MD trajectories. The red line indicates a standardized normal distribution. **B** Probability distribution of the noise variance $\sigma^2_{\mathrm{n},p}$ for the trajectories considered in **A**. The red dashed vertical line indicates the mean noise variance of the selected samples. The standard deviation of the noise variance is less than 5% of the mean, which legitimates the homoscedastic noise assumption. **C** Same as **A** but for wall shear stress τ. **D** Same as **B** but for the noise variance of the wall shear stress $\sigma^2_{\mathrm{n},\tau}$.
